# Disulfiram modulated ROS–MAPK and NF*κ*B pathways and targeted breast cancer cells with cancer stem cell-like properties

**DOI:** 10.1038/bjc.2011.126

**Published:** 2011-04-12

**Authors:** N C Yip, I S Fombon, P Liu, S Brown, V Kannappan, A L Armesilla, B Xu, J Cassidy, J L Darling, W Wang

**Affiliations:** 1 Research Institute in Healthcare Science, School of Applied Sciences, University of Wolverhampton, Wolverhampton WV1 1LY, UK; 2 Department of Heamatology, Nanfang Hospital, The Southern Medical University, Guangzhou, People's Republic of China; 3 Department of Medical Oncology, Beatson Institute for Cancer Research, Glasgow, UK

**Keywords:** disulfiram, reactive oxygen species, NF*κ*B, breast cancer stem cells, paclitaxel, MAPK pathway

## Abstract

**Background:**

Previous studies indicate that disulfiram (DS), an anti-alcoholism drug, is cytotoxic to cancer cell lines and reverses anticancer drug resistance. Cancer stem cells (CSCs) are the major cause of chemoresistance leading to the failure of cancer chemotherapy. This study intended to examine the effect of DS on breast cancer stem cells (BCSCs).

**Methods:**

The effect of DS on BC cell lines and BCSCs was determined by MTT, western blot, CSCs culture and CSCs marker analysis.

**Results:**

Disulfiram was highly toxic to BC cell lines *in vitro* in a copper (Cu)-dependent manner. In Cu-containing medium (1 *μ*M), the IC_50_ concentrations of DS in BC cell lines were 200–500 nM. Disulfiram/copper significantly enhanced (3.7–15.5-fold) cytotoxicity of paclitaxel (PAC). Combination index isobologram analysis demonstrated a synergistic effect between DS/Cu and PAC. The increased Bax and Bcl2 protein expression ratio indicated that intrinsic apoptotic pathway may be involved in DS/Cu-induced apoptosis. Clonogenic assay showed DS/Cu-inhibited clonogenicity of BC cells. Mammosphere formation and the ALDH1^+VE^ and CD24^Low^/CD44^High^ CSCs population in mammospheres were significantly inhibited by exposure to DS/Cu for 24 h. Disulfiram/copper induced reactive oxygen species (ROS) generation and activated its downstream apoptosis-related cJun N-terminal kinase and p38 MAPK pathways. Meanwhile, the constitutive NF*κ*B activity in BC cell lines was inhibited by DS/Cu.

**Conclusion:**

Disulfiram/copper inhibited BCSCs and enhanced cytotoxicity of PAC in BC cell lines. This may be caused by simultaneous induction of ROS and inhibition of NF*κ*B.

The development of drug resistance remains the major obstacle to the success of breast cancer (BC) chemotherapy. Drug-induced DNA damage triggers the expression of anti-apoptotic proteins, which confer drug resistance upon cancer. The NF*κ*B is one of the major chemoresistance-related anti-apoptotic factors. Many human cancers including BC possess high levels of the constitutive NF*κ*B activity, which can be further induced by some anticancer drugs. High NF*κ*B activity links inflammation and tumourigenesis (Greten *et al*, 2004). Activated NF*κ*B triggers a series of molecular reactions including up-regulation of anti-apoptotic protein-encoding genes (Dutta *et al*, 2006) that induce cancer chemoresistance. High NF*κ*B activity has been identified in drug-resistant cancer cells and ectopic over-expression of NF*κ*B can block anticancer drug-induced apoptosis (Wang *et al*, 1998, 1999, 2004). Previous studies in our laboratory demonstrate that 5-fluorouracil (5-FU)- and gemcitabine (dFdC)-resistant cancer cell lines possess higher NF*κ*B activity (Wang and Cassidy, 2003; Guo *et al*, 2009). Over-expression of p50 and p65, the two subunits of NF*κ*B, results in increased NF*κ*B activity and induces 5-FU and dFdC resistance (Wang *et al*, 2004; Guo *et al*, 2009). Although NF*κ*B is an attractive molecular target for therapeutic intervention, inhibition of NF*κ*B alone can only induce limited cell death. The disappointing clinical trial outcomes from using NF*κ*B inhibitor in treatment of metastatic BC patients (Yang *et al*, 2006; Cresta *et al*, 2008) indicate that BC chemotherapy cannot be efficiently improved by only targeting NF*κ*B pathway.

Reactive oxygen species (ROS) (Gupte and Mumper, 2009) are a group of oxygen-containing chemical species normally generated from mitochondrial respiratory chain reaction with reactive chemical properties. High ROS activity can damage DNA, protein and lipid membrane leading to apoptosis. In comparison with normal tissues, cancer cells generally possess high ROS activity (Fruehauf and Meyskens, 2007) and can tolerate higher levels of ROS. It has been suggested that further increasing ROS exposure induced by ROS-generating agents will exhaust the cellular antioxidant capacity, pushing cancer cells over the tolerated ROS threshold and leading to apoptosis (Lopez-Lazaro, 2007). Reactive oxygen species-induced apoptosis is highly reliant on persistent activation of pro-apoptotic MAPK pathways (cJun N-terminal kinases (JNKs) and p38) (Nakano *et al*, 2006) mainly through modulating the activities of mitochondrial pro- and anti-apoptotic proteins by phosphorylation events (Junttila *et al*, 2008). Many conventional anticancer drugs induce ROS generation and trigger cancer cell apoptosis via ROS–MAPK pathway. However, anticancer drug-induced ROS activation also triggers expression and activation of a number of anti-apoptotic factors including NF*κ*B that dampen the ROS-induced cytotoxic effect (Nakano *et al*, 2006).

Owing to the cross-talk between NF*κ*B and ROS–MAPK pathways, singly targeting either pathway may not be sufficient for inducing cancer cell killing. Therefore, identification of small molecules that simultaneously activate the ROS–MAPK pro-apoptotic pathway and block ROS-induced anti-apoptotic pathways may improve BC chemotherapy. Disulfiram (DS) is a commercially available anti-alcoholism drug (Johansson, 1992). We have previously demonstrated that DS inhibits NF*κ*B activity and enhances 5-FU- and dFdC-induced apoptosis in drug-sensitive and -resistant colon cancer cell lines (Wang *et al*, 2003; Guo *et al*, 2009). Disulfiram also potentiates the cytotoxicity of cyclophosphamide, cisplatin and radiation *in vitro* and protects normal cells in kidney, gut and bone marrow *in vivo*, while increasing the therapeutic index of a wide range of cytotoxic drugs (Evans *et al*, 1982; Hacker *et al*, 1982; Bodenner *et al*, 1986). The molecular anticancer mechanisms of DS are still largely unknown. The previous publications indicate that the anticancer effect of DS is copper (Cu) dependent (Nobel *et al*, 1995; Cen *et al*, 2002, 2004; Chen *et al*, 2006). Copper has a crucial role in redox reactions and triggers generation of ROS in human cells. Disulfiram/copper is a strong ROS inducer (Nobel *et al*, 1995) and proteasome-NF*κ*B pathway inhibitor (Chen *et al*, 2006). Combination of DS with Cu may target cancer cells by simultaneously tackling both ROS and NF*κ*B.

Cancer derives from a very small fraction (1%) of cancer stem cells (CSCs) (Dalerba *et al*, 2007), which are relatively quiescent and express multidrug resistant and anti-apoptotic proteins (Marques *et al*, 2010; Storci *et al*, 2010). Conventional anticancer drugs target the proliferating and differentiated tumour bulk, but fail to eradicate the CSCs, which become the source of tumour recurrence. Aldehyde dehydrogenases (ALDHs) are functional markers of normal and breast cancer stem cells (BCSCs) (Ginestier *et al*, 2007; Marcato *et al*, 2011). It recently reported that targeting ALDH1A1 gene can target ovarian CSCs (Landen *et al*, 2009). Disulfiram is a specific inhibitor of ALDHs (Johansson, 1992; Lam *et al*, 1997). Therefore, it may also be an inhibitor of BCSCs.

This study demonstrated that in combination with physiological concentration of Cu, DS was highly cytotoxic to BCSCs and synergistically enhanced the cytotoxicity of paclitaxel (PAC) in BC cell lines.

## Materials and methods

### Cell lines and reagents

The BC cell lines MCF7, MDA-MB-231 and T47D were purchased from ATCC (Teddington, UK). Disulfiram, copper (II) chloride (CuCl_2_), *N*-acetyl-cysteine (NAC), SP600125 and SB203580 were purchased from Sigma (Dorset, UK).

### Cell culture and cytotoxicity analysis

All cell lines were cultured in DMEM (Lonza, Wokingham, UK) supplemented with 10% FCS, 50 units ml^–1^ penicillin and 50 *μ*g ml^–1^ streptomycin. For *in vitro* cytotoxicity assay, cells (5000 per well) were cultured overnight in 96-well flat-bottomed microtitre plates, then exposed to drugs for 72 h and subjected to a standard MTT assay (Plumb *et al*, 1989).

### Analysis of the combinational effect of PAC and DS/Cu by combination index isobologram

Overnight cultured cells were exposed to various concentrations of PAC, DS+Cu_1 *μ*M_ or in combination of PAC and DS+Cu_1 *μ*M_ at a constant PAC:DS ratio of 62.5:1 for 72 h. The cells were then subjected to MTT analysis as described above. The combinational cytotoxicity of PAC and DS/Cu_1*μ*M_ was determined using combination index (CI) isobologram analysed by CalcuSyn software (Biosoft, Cambridge, UK) (Chou and Talalay, 1984). The CI was determined by mutually exclusive equations.

### Western blot analysis

Cells (80% confluence) were collected by trypsinisation and washed in ice-cold PBS and lysed in RIPA buffer. The lysate was centrifuged for 5 min in a microfuge and the supernatants retained. The primary antibodies (Cell Signaling, Herts, UK: JNK, phosphorylated JNK, cJun, phosphorylated cJun, phosphorylated p38 and cleaved PARP; Santa Cruz, CA, USA: Bcl2 and Bax) were diluted at 1:1000 in 3% BSA-TBST (anti-phosphorylated protein) or 5% fat-free milk-TBST (anti-non-phosphorylated protein). Anti-*α*-tubulin (Amersham, Buckinghamshire, UK; 1:8000 diluted) was used as loading control. The signal was detected using an ECL western blotting detection kit (GeneFlow, Staffordshire, UK).

### Electrophoretic mobility-shift assays

Detection of NF*κ*B-oligonucleotide complex was performed using a LightShift chemiluminescent electrophoretic mobility-shift assay kit (Pierce, Northumberland, UK). Briefly, nuclear protein (5 *μ*g) was incubated with 20 fmol of biotin-labelled oligonucleotide for 20 min at room temperature in binding buffer. The specificity of the NF*κ*B DNA binding was determined in competition reactions in which a 200-fold molar excess of unlabelled wild-type (5′-AGT TGA GGG GAC TTT CCC AGG C-3′) or mutant (5′-AGT TGA TAT TAC TTT TAT AGG C-3′) NF*κ*B probes were added to the binding reaction. The signal was detected by chemiluminescent photography.

### Flow cytometric analysis of DNA content

Cells (1 × 10^6^) were exposed to drugs and harvested by trypsinisation. The cells were fixed in 70% ethanol and then incubated with RNase A (100 *μ*g ml^–1^) and propidium iodide (50 *μ*g ml^–1^) for 30 min. The data from 10 000 cells of each sample were collected by FACS Scan (Becton Dickinson, Franklin Lakes, NJ, USA) and the DNA content analysed using CellQuest software (BD Biosciences, Oxford, UK).

### ROS activity detection

The intracellular ROS levels were determined using 2′,7′-dichlorodihydrofluorescein diacetate (H_2_DCFDA) probe (Invitrogen, Paisley, UK). Cancer cells (1 × 10^6^) were cultured in 24-well plates with 1 ml of serum- and phenol red-free DMEM medium (Sigma) containing 20 *μ*M of H_2_DCFDA. Fluorescence was measured in 96-well plates at excitation 490 nm and emission 520 nm using a Fluoroskan Ascent fluorometer (Thermo Scientific, Northumberland, UK).

### Luciferase reporter gene assay

All the transfections were performed using Lipofectamine 2000 (Invitrogen) transfection reagent following the manufacturer's instructions. The cells were co-transfected with luciferase reporter vectors (pNF*κ*B-Tal-Luc (BD Biosciences) and pGL3-Basic (Promega, Southampton, UK)) and an internal control, pSV40-Renilla (Promega). The luciferase activities were determined using Dual Luciferase Assay reagents (Promega) following the manufacturer's instructions. The luciferase activity in each well was normalised to pSV40-Renilla using the formula of Ln=*L*/*R* (Ln, normalised luciferase activity; *L*, luciferase activity reading and *R*, Renilla activity reading). The Ln was further standardised by the transcriptional activity of the pGL3-Basic using the formula of RLU = Ln_NF_*κ*_B_/Ln_Basic_ (RLU, relative luciferase unit).

### Clonogenic assay

Cells (5 × 10^4^ per well in six-well plates) were exposed to designated concentration of DS/Cu_1 *μ*M_, PAC or PAC+DS/Cu_1 *μ*M_ for 24 h. The cells were collected and further cultured for 7 (MDA-MB-231 and MCF7) to 14 (T47D) days in six-well plates containing drug-free medium at a cell density of 2.5 × 10^3^ per well. Clonogenic cells were determined as those able to form a colony consisting of at least 50 cells.

### Detection of ALDH-positive population

The ALDH-positive population in drug-treated BC cell lines was detected by ALDEFLUOR kit (StemCell Tech., Durham, NC, USA) following the supplier's instruction. The cells (2.5 × 10^5^) were analysed after staining in ALDH substrate containing assay buffer for 30 min at 37°C. The negative control was treated with diethylaminobenzaldehyde (DEAB), a specific ALDH inhibitor.

### *In vitro* mammosphere culture

The BC cells were cultured in ultra-low adherence six-well plates (Corning, Woburn, MA, USA) containing 2 ml of stem cell culture medium (SCM, serum-free DMEM-F12 supplemented with B27 (Invitrogen), 20 ng ml^–1^ epidermal growth factor (Sigma), 10 ng ml^–1^ basic fibroblasts growth factor (R & D System, Abingdon, UK), 10 *μ*g ml^–1^ insulin (Sigma)) at a density of 10 000 cells ml^–1^. After 7–10 days culture, the mammospheres were photographed and subjected to further treatments.

### Flow cytometric analysis of CD24 and CD44 expression

The adherent or mammosphere cells were trypsinised and passed through a 25G needle. The cells (2.5 × 10^5^) were incubated with CD24 and CD44 antibodies (BD Pharmingen, Oxford, UK) for 20 min at 4°C. Unbound antibodies were washed off with 2% FCS HBSS (Sigma) and the cells (10 000 events) were examined no longer than 1 h after staining on a BD Facscalibur (Dorset, UK).

### Statistical analysis

The data analysis was performed using Student's *t*-test and one-way ANOVA.

## Results

### The cytotoxicity of DS in BC cells was Cu dependent

**Figure 1:**
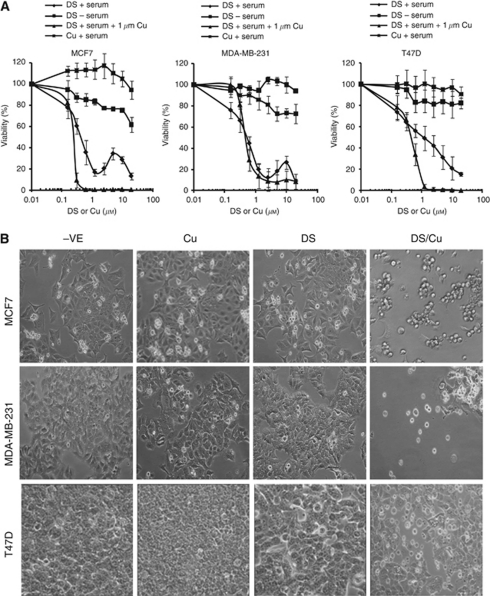

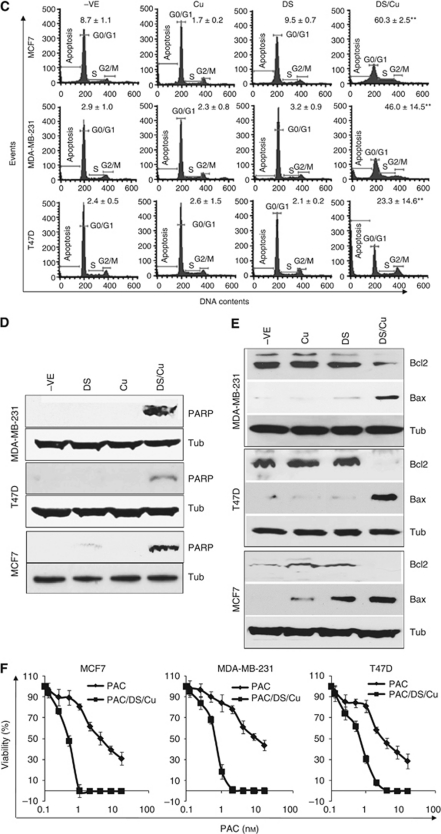

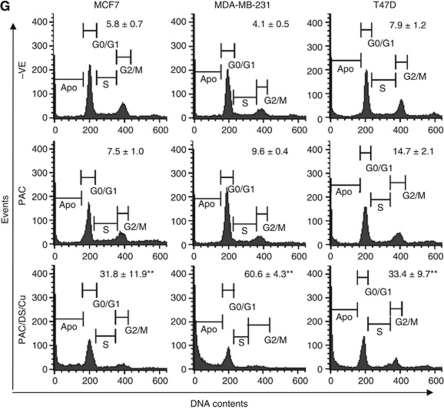
Disulfiram was cytotoxic to BC cells in a copper-dependent manner and synergistically enhanced cytotoxicity of PAC in BC cell lines. (**A**) MTT cytotoxicity assay. The BC cells were exposed to different treatments for 72 h. (**B**) The morphology ( × 100 magnification) of BC cell lines after 72 h drug exposure (DS: 1 *μ*M of DS in serum-free medium, Cu: CuCl2 1 *μ*M, DS/Cu: DS 1 *μ*M + Cu 1 *μ*M). (**C**) The DNA contents of BC cells after 72 h drug exposure (DS: 1 *μ*M of DS in serum-free medium, Cu: CuCl2 1 *μ*M, DS/Cu: DS 1 *μ*M+Cu 1 *μ*M). The DNA contents in the treated cells (10 000 events) were determined. The sub-G1 population represents the apoptotic cells (^**^P<0.01, *n*=3). The cleavage of PARP protein (**D**) and the expression levels of Bcl2 and Bax (**E**) after 72 h drug exposure were determined by western blot. Tub: *α*-tubulin used as a loading control. (**F**) MTT analysis of the combined effect of PAC and DS/Cu_1 *μ*M_. PAC:DS/Cu_1 *μ*M_=1:62.5. (**G**) The PAC-induced apoptosis was enhanced by DS/Cu. The DNA contents in the cell lines treated for 72 h with PAC (1 nM) or PAC plus DS/Cu (DS: MCF7, 100 nM; MDA-MB-231 and T47D, 150 nM; Cu: CuCl2 1 *μ*M) were determined by flow cytometry. (^**^P<0.01, *n*=3).

In CuCl_2_ (1 *μ*M)-supplemented medium, DS was highly cytotoxic to BC cell lines (IC_50_72h_: 110–476 nM; Figure 1A; Table 1). Disulfiram was also toxic to cancer cell lines in the complete medium without CuCl_2_ supplement with higher IC_50s_ (456–1100 nM; Figure 1A; Table 1). A biphasic effect was observed in two out of three BC cell lines. The cancer cells appeared to be protected at higher concentrations of DS. Disulfiram alone in serum-free medium (to rule out the influence of trace amount of Cu contained in FCS) or Cu alone was not toxic to BC cell lines even at a very high concentration (20 *μ*M). The drug-induced morphological changes are shown in Figure 1B. The flow cytometric DNA content analysis demonstrated significant increase of apoptosis (sub-G1 population) in 72 h DS/Cu treated, but not other groups (Figure 1C). The cleaved PARP protein, an indicator of caspase activation, was detected in DS/Cu-treated cells (Figure 1D). Disulfiram/copper significantly inhibited Bcl2 and induced Bax expression; therefore, the Bax/Bcl2 ratio was increased in DS/Cu-treated cells (Figure 1E).

### DS/Cu synergistically enhanced the cytotoxicity of PAC in BC cell lines

In combination with DS/Cu, the cytotoxicity of PAC was significantly enhanced in BC cell lines (4–16-fold) (Figure 1F; Table 1). There was a very strong synergistic effect between DS/Cu and PAC over a wide range of concentrations (IC_50_–IC_90_; Table 1). In contrast to the slight induction of apoptosis at low concentration of PAC alone (1 nM), the proportion of apoptotic cells was massively increased by DS/Cu (DS 100–150 nM/Cu 1 *μ*M) and PAC in combination (Figure 1G).

### ROS activation was responsible for DS/Cu-induced cytotoxicity

**Figure 2:**
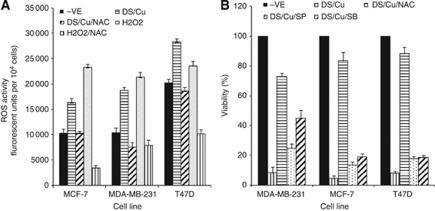
ROS was responsible for DS/Cu-induced cytotoxicity. (**A**) The BC cell lines were loaded with fluorescent dye H_2_DCFDA and exposed to DS_5 *μ*M_/Cu_5 *μ*M_ or DS/Cu plus NAC (10 mM) for 3 h. The fluorescent strength was detected by fluorometer at Ex 490 nm and Em 520 nm. (**B**) The effect of ROS and MAPK pathway inhibitors on DS/Cu-induced cytotoxicity. The cancer cells were exposed to DS_250 nM_/Cu_1 *μ*M_, DS/Cu plus NAC (10 mM), SP600125 (10 *μ*M) or SB203580 (10 *μ*M) for 72 h and subjected to MTT assay.

The Cu-dependent cytotoxicity of DS indicates that ROS may be the mediator for DS/Cu-induced apoptosis. Disulfiram/copper significantly induced ROS activity in BC cell lines (*P*<0.01), which was reversed by addition of an ROS inhibitor, NAC (*P*<0.01; Figure 2A). To determine the effect of ROS on DS/Cu-induced cell death, the cytotoxicity assay was performed with or without ROS inhibitor. As shown in Figure 2B, the DS/Cu-induced cytotoxicity was significantly reversed by addition of NAC in the culture (*P*<0.01).

### DS/Cu triggered persistent activation of JNK and p38 pathways

**Figure 3:**
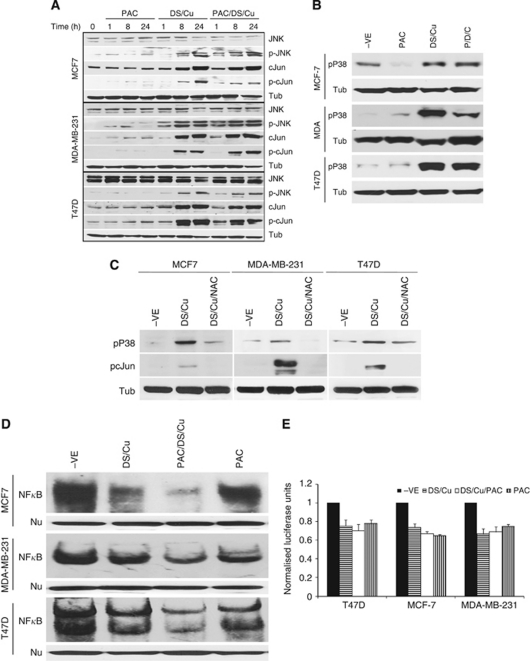
The effect of DS/Cu on MAPK and NF*κ*B pathways. The overnight cultured BC cells were exposed to PAC_1 *μ*M_, DS_1 *μ*M_/Cu_1 *μ*M_ or PAC_1 *μ*M_/DS_1 *μ*M_/Cu_1 *μ*M_ for indicated time lengths. The expression levels and phosphorylation status of proteins in JNK (**A**) and p38 (**B**) pathways were detected by western blot. (**C**) The activation of JNK and p38 pathways was reversed by NAC. The phosphorylation of cJun and p38 in BC cell lines was determined by western blot after exposure to DS/Cu or DS/Cu plus NAC (10 mM) for 24 h. (**D**) NF*κ*B DNA-binding activity was analysed by electrophoretic mobility-shift assay assay. Nu: western blot of nucleolin was used as a protein loading control. The BC cell lines were treated with PAC_1 *μ*M_, DS_1 *μ*M_/Cu_1 *μ*M_ or PAC/DS/Cu for 24 h. (**E**) NF*κ*B transcriptional activity examined by luciferase reporter gene assay after exposure to PAC_1 *μ*M_, DS_1 *μ*M_/Cu_1 *μ*M_ or PAC/DS/Cu for 24 h.

Figure 3A shows the effect of PAC, DS/Cu and PAC/DS/Cu on the activation of the JNK pathway. Total JNK protein expression was not affected by the above treatments. However, the expression of phosphorylated JNK, cJun and total cJun was persistently (up to 24 h) induced by DS/Cu and PAC/DS/Cu. In contrast, the expression of these proteins was not or only very mildly up-regulated by PAC. High levels of phosphorylated p38 were also detected up to 24 h following DS/Cu and PAC/DS/Cu exposure (Figure 3B). To determine the causal relationship between ROS and JNK, p38 pathways, BC cell lines were exposed to DS/Cu for 24 h with or without addition of NAC. *N*-acetyl-cysteine significantly inhibited or totally blocked DS/Cu-induced cJun and p38 phosphorylation (Figure 3C). cJun N-terminal kinase and p38 are the major pathways responsible for ROS-induced apoptosis (McCubrey *et al*, 2006). Singly blocking JNK or p38 also reversed the DS/Cu-induced cytotoxicity, but at a significantly lower levels than NAC-induced ROS blocking (*P*<0.01; Figure 2B).

### DS/Cu inhibited NF*κ*B activity in BC cell lines

The NF*κ*B is an ROS-induced transcription factor with strong anti-apoptotic activity, which in turn dampens the pro-apoptotic effect of ROS (Nakano *et al*, 2006). Blockage of NF*κ*B activation enhances ROS-induced cytotoxicity. Both PAC and DS/Cu inhibited NF*κ*B DNA-binding activity in BC cell lines. The strongest inhibition was observed in the cells treated with PAC/DS/Cu in combination (Figure 3D). The inhibition of NF*κ*B transcriptional activity was also detected in PAC-, DS/Cu- and PAC/DS/Cu-treated cells by reporter gene assay (Figure 3E).

### DS/Cu inhibited the clonogenity in BC cell lines

**Figure 4:**
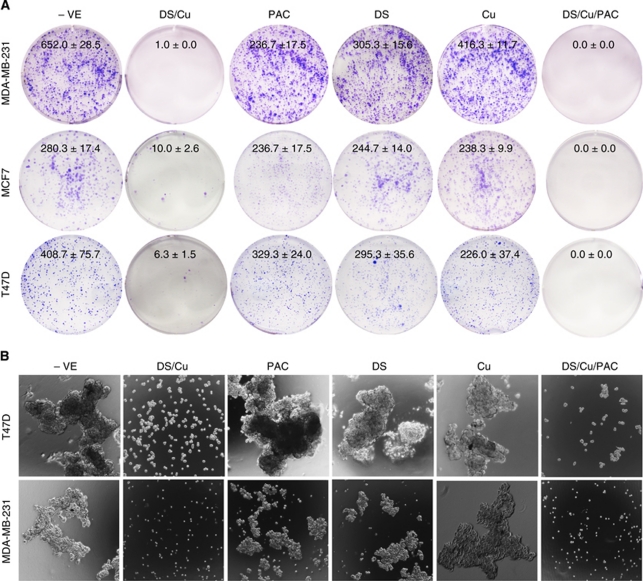

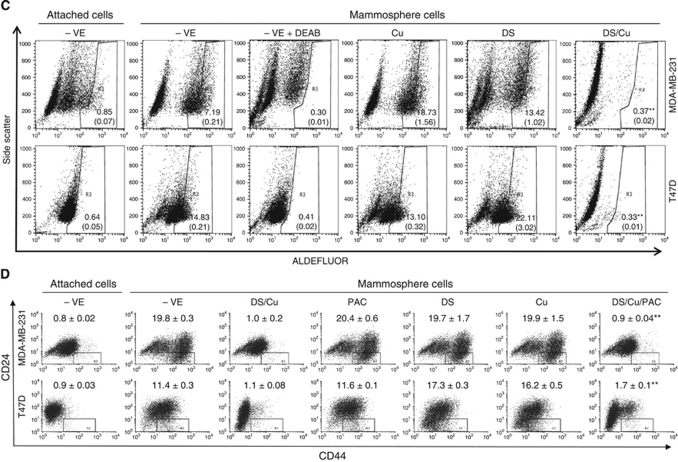
The effect of DS/Cu on the clonogenicity and CSCs in BC cell lines. (**A**) Clonogenic assay. The cells exposed to Cu_1 *μ*M_, PAC_40 nM_, DS (250 nM for MDA-MB-231 and T47D, 200 nM for MCF7), DS/Cu or PAC/DS/Cu for 24 h were cultured in drug-free medium in six-well plates at a cell density of 2.5 × 10^3^ per well for 7–10 days. The colonies were stained with crystal violet, counted and photographed as described in Materials and Methods. (**B**) DS/Cu and PAC/DS/Cu inhibited mammosphere formation. The BC cells were treated with PAC_40 nM_, DS_250 nM_, Cu_1 *μ*M_, DS/Cu or PAC/DS/Cu for 48 h and then sub-cultured in drug-free SCM in ultra-low attachment six-well plates (5000 cells per well) for 7 days and photographed at × 40 magnification. (**C**) DS/Cu inhibited ALDH expression in mammospheres. The ALDH^+VE^ population was flow cytometrically determined in mammospheres exposed to drugs (Cu_1 *μ*M_, DS_1 *μ*M_ or DS_1 *μ*M_/Cu_1 *μ*M_) for 16 h. (**D**) DS/Cu abolished CD24^Low^/CD44^High^ population in mammospheres. The expression of CD24 and CD44 was examined after 16 h exposure to Cu_1 *μ*M_, DS_1 *μ*M_, PAC_100 nM_ or DS/Cu/PAC. The inserted numbers in the frame represent percentage of ALDH^+VE^ or CD24^Low^/CD44^High^ cells (mean±s.d. from three experiments, ^**^P<0.01).

Clonogenic assays (Franken *et al*, 2006) were performed to examine the ability of DS/Cu to induce ‘reproductive death’ in BC cells. After 16 h exposure to PAC (40 nM: 4–18-fold higher than IC_50_ concentration), DS (200–250 nM: sub-IC_50_ concentration)/Cu_1 *μ*M_ or PAC and DS/Cu in combination, the treated cells were collected and cultured in drug-free medium for 7–14 days. The colony number was reduced by exposure to PAC, DS or Cu alone. The colony number in PAC-, DS- and Cu-treated groups was decreased, which was caused by slow growth of the surviving cells leading to the cell number in some colonies not reaching the counting threshold (50 cells). In contrast, the clonogenicity of BC cell lines was significantly inhibited by DS/Cu and totally eradicated by exposure to PAC plus DS/Cu (Figure 4A).

### DS/Cu targeted BCSCs

Furthermore, we examined the effect of different treatments on CSCs population in MDA-MB-231 and T47D cell lines. The mammosphere formation in both cell lines was completely blocked by exposure to DS_1 *μ*M_/Cu_1 *μ*M_ or DS/Cu plus PAC_40 nM_ for 48 h, but not affected by PAC, DS or Cu alone (Figure 4B). To determine the targeting effect of DS/Cu on CSCs, the BCSCs markers in drug-treated mammosphere cells were also analysed. Figure 4C demonstrates that the ALDH-positive population in mammospheres was significantly inhibited by DS/Cu, but not affected or even enriched by DS or Cu treatment. In order to determine the effect of DS/Cu on CSCs, the expression status of CD24^Low^/CD44^High^, another marker of BCSCs, was also examined. After 16 h exposure to different drugs, the CD24^Low^/CD44^High^ population in the mammosphere cells was determined by flow cytometry. In comparison with the attached cells, the mammosphere population contained significantly higher percentage of CD24^Low^/CD44^High^ BCSCs (Figure 4D). The percentage of CD24^Low^/CD44^High^ cells in mammosphere was significantly reduced following 16 h exposure to DS/Cu and PAC/DS/Cu, but not influenced by PAC, DS or Cu (Figure 4D).

## Discussion

Disulfiram is a Food and Drug Administration-approved anti-alcoholism drug used in clinic with extensive available pre-clinical and clinical data (Eneanya *et al*, 1981). Our study demonstrated high cytotoxicity of DS to BC cell lines in a Cu-dependent manner. Using Cu to treat cancer has a long history (Hieger, 1926; Gupte and Mumper, 2009), but the intracellular transport of Cu is still one of the major hurdles for its clinical efficacy. The transport of Cu into cell is mediated and tightly controlled by the copper transporter, Ctr1. A derivative of DS, *N*,*N*-diethyldithiocarbamate (deDTC), binds to Cu forming a Cu(deDTC)_2_ complex, which improves the intracellular trafficking of Cu and this is probably responsible for DS-induced apoptosis (Cen *et al*, 2004). Disulfiram can also penetrate into cancer cells to form Cu(deDTC)_2_ with intracellular Cu. In comparison with normal tissues, many cancers including BC possess higher levels of Cu (two- to three-fold) (Mulay *et al*, 1971; Margalioth *et al*, 1983; Rizk and Sky-Peck, 1984), which may enable DS to target cancer cells selectively (Evans *et al*, 1982; Hacker *et al*, 1982; Bodenner *et al*, 1986; Chen *et al*, 2006; Iljin *et al*, 2009). In line with a previous report (Wickstrom *et al*, 2007), a biphasic cytotoxic effect of DS was observed in BC cell lines tested in complete medium without Cu supplement (Figure 1A). Breast cancer cells were killed at low concentration, but revived at higher DS concentrations (∼10 *μ*M). The mechanism of the biphasic effect remains unclear. A degradation product of DS may compete trace amounts of Cu, block formation of Cu(deDTC)_2_ and inhibit transport of Cu into cells (Cen *et al*, 2004). We have previously reported that DS enhances the cytotoxicity of 5-FU and gemcitabine in colon and BC cell lines (Wang *et al*, 2003; Guo *et al*, 2009). Here, we demonstrated synergistic cytotoxic effect of DS/Cu and PAC on BC cell lines.

Previous studies demonstrate that in combination with Cu, DS induces ROS activity in melanoma cell lines (Cen *et al*, 2002; Morrison *et al*, 2010). The recent study from Dou's group demonstrates that gold–dithiocarbamato complexes strongly induce ROS and inhibit proteasome activity in BC cells (Zhang *et al*, 2010). In consistence with these results, our study showed that DS/Cu induced ROS activity, which was responsible for DS/Cu-induced cytotoxicity in BC cell lines. The ROS-induced apoptosis is commonly mediated by the persistent activation of JNK and p38 MAPK pathways (Junttila *et al*, 2008). In our study, both JNK and p38 pathways were persistently (over 24 h) activated (phosphorylation of cJun and p38) by DS/Cu and blocked by NAC. cJun N-terminal kinase and p38 inhibitors reduced cytotoxicity of DS/Cu, although to a lesser degree than ROS inhibition. Therefore, ROS-activated JNK and p38 pathways were, at least partially, responsible for ROS-induced apoptosis. The persistent activation of JNK and p38 induces apoptosis via mitochondrial apoptotic pathways (Junttila *et al*, 2008). The DS/Cu-induced apoptosis was confirmed by DNA content and PARP protein cleavage. The expression of Bax and Bcl2 proteins was induced and suppressed by DS/Cu, respectively, leading to increased Bax/Bcl2 ratio. The elevated Bax/Bcl2 ratio indicated that the intrinsic apoptotic pathway may be involved in DS/Cu-induced apoptosis.

Owing to the high proliferative rate and energy requirement, cancer cells are under higher ROS stress than their normal counterparts. High levels of ROS can damage DNA, mitochondrial inner membrane and membrane phospholipids leading to apoptosis (Gupte and Mumper, 2009). However, ROS also activate a wide range of anti-apoptotic factors. The effect of ROS on cancer cells depends on the balance between ROS-induced pro- and anti-apoptotic factors. The NF*κ*B is one of the most important ROS-induced anti-apoptotic factors (Gloire *et al*, 2006). The NF*κ*B activation in turn inhibits ROS and JNK, p38 activation and ultimately inhibits ROS-induced apoptosis. Breast cancer cell lines commonly possess high levels of constitutive NF*κ*B activity (Wang *et al*, 2004; Guo *et al*, 2009; Xu *et al*, 2009). Consistent with previous publications (Wang *et al*, 2003; Guo *et al*, 2009), DS/Cu inhibited NF*κ*B activity in BC cell lines. This indicates that DS/Cu may induce apoptosis of BC cells by simultaneously inducing ROS generation and inhibiting ROS-NF*κ*B pathway.

The effect of DS/Cu on the regeneration of minimal-residual cancer cells, the main source of cancer relapse after chemotherapy, was examined using a clonogenic assay, a gold measure to detect the cell ‘reproductive death’ after cytotoxic agent treatments (Franken *et al*, 2006). In contrast to the moderate inhibiting effect of PAC, DS and Cu on clonogenicity of BC cells, the colony formation was significantly reduced or completely eradicated by DS/Cu and PAC/DS/Cu, respectively (Figure 4A).

Disulfiram/copper reverses cancer cell chemoresistance induced by a wide range of different mechanisms (Wang *et al*, 2001, 2003, 2004; Guo *et al*, 2009). It has been widely accepted that CSCs are responsible for tumour recurrence and may display significant resistance to different cytotoxic drugs (Liu and Wicha, 2010). The effect of DS/Cu on clonogenicity of BC cell lines prompted us to examine the effect of DS/Cu on BCSCs. Disulfiram is an inhibitor of ALDHs. Human ALDHs are a superfamily with 19 members involved in detoxifying a wide range of aldehydes to their corresponding weak carboxylic acids (Sladek, 2003); ALDH1A1 has been identified as a functional marker of several different types of CSCs including BCSCs (Ginestier *et al*, 2007; Alison *et al*, 2011). It recently reported that knockdown of ALDH1A1 expression using siRNA can target ovarian CSCs and potentiate cytotoxicity of taxane and platinum *in vitro* and *in vivo* (Landen *et al*, 2009). Recently, Marcato *et al* (2011) identify ALDH1A3 as another major marker of BCSCs. Therefore, ALDHs may be redundantly expressed in different cancer types and targeting one isoform may not be sufficient for CSCs targeting. Disulfiram is a strong inhibitor for both cytosol and mitochondrial ALDHs (Eneanya *et al*, 1981; Lam *et al*, 1997). Kast and Belda-Iniesta (2009) hypothesised that targeting ALDHs by DS may reverse chemoresistance in glioblastoma. Our study is the first report of using DS to target BCSCs. The ALDH^+VE^ population in BCSCs was significantly inhibited by DS/Cu. The ability of BC cell lines to form mammospheres was completely inhibited by 24 h exposure to DS/Cu or PAC/DS/Cu (Figure 4B). The effect of DS/Cu on CSCs was also confirmed by the reduction of the CD24^Low^/CD44^High^ population (Figures 4C and D). The detailed molecular mechanisms underlying the effect of DS/Cu on BCSCs are unclear. Aldehyde dehydrogenases detoxify intracellular aldehydes, which can form adducts with glutathione, nucleic acids and amino acids leading to cell death (Marchitti *et al*, 2008). The high expression of ALDHs in CSCs may be protective. Mammalian cornea cells contain abundant ALDH, which has critical role in scavenging ROS and reduce UV-induced oxidative stress (Estey *et al*, 2007). Aldehyde dehydrogenase deficiency in central nervous system is associated with progressive neurodegeneration (Marchitti *et al*, 2007). Inhibition of NF*κ*B pathway and induction of ROS result in reduction of stem-like properties in CSCs derived from pancreatic cancer and leukaemia (Greten *et al*, 2004; Jin *et al*, 2010; Rausch *et al*, 2010). Disulfiram/copper may target BCSCs by simultaneously inhibiting NF*κ*B and activating ROS activity.

## Figures and Tables

**Table 1:** Cytotoxicity of different treatments to breast cancer cell lines

	**MCF7**	**MDA-MB-231**	**T47D**
*IC*_*50*_ *(nM)*			
DS+serum	456 (62)	495 (49)	1100 (87)
DS/Cu	211 (23)	476 (48)	443 (62)
DS–serum	>20 000	>20 000	>20 000
Cu	>20 000	>20 000	>20 000
PAC alone	4.3 (1.4)	9.3 (0.7)	2.6 (0.3)
PAC+DS/Cu	0.4^**^ (0.1)	0.6^**^ (0.02)	0.7^**^ (0.1)
			
*CI values*			
IC_50_	0.183	0.437	0.446
IC_75_	0.213	0.436	0.661
IC_90_	0.265	0.457	0.633

Abbreviations: CI=combination index; Cu=copper; DS=disulfiram; PAC=paclitaxel.

The figure represents IC_50_ value from three experiments (mean (s.d.)). ^**^Compared with PAC alone, significant difference (*P*<0.01, *n*=3). The cells were treated for 72 h. DS/Cu: DS in medium supplemented with 1 *μ*M CuCl_2_; DS–serum: DS in serum-free medium; DS+serum: DS in serum-containing medium.
